# Intertidal marine sediment harbours Actinobacteria with promising bioactive and biosynthetic potential

**DOI:** 10.1038/s41598-017-09672-6

**Published:** 2017-08-30

**Authors:** Polpass Arul Jose, Bhavanath Jha

**Affiliations:** 1 Marine Biotechnology and Ecology Division, CSIR- Central Salt and Marine Chemicals Research Institute, G. B. Marg, Bhavnagar - 364002, Gujarat India; 2 Academy of Scientific and Innovative Research (AcSIR), Council of Scientific and Industrial Research (CSIR), New Delhi, India

## Abstract

Actinobacteria are the major source of bioactive natural products that find their value in research and drug discovery programmes. Antimicrobial resistance and the resulting high demand for novel antibiotics underscore the need for exploring novel sources of these bacteria endowed with biosynthetic potential. Intertidal ecosystems endure regular periods of immersion and emersion, and represent an untapped source of Actinobacteria. In this study, we studied the diversity and biosynthetic potential of cultivable Actinobacteria from intertidal sediments of Diu Island in the Arabian Sea. A total of 148 Actinobacteria were selectively isolated using a stamping method with eight isolation media. Isolates were grouped into OTUs based on their 16S rRNA gene sequence, and categorized within actinobacterial families such as Glycomycetaceae, Micromonosporaceae, Nocardiaceae, Nocardiopsaceae, Pseudonocardiaceae, Streptomycetaceae, and Thermomonosporaceae. The biosynthetic potential of the Actinobacteria, necessary for secondary metabolite biosynthesis, was screened and confirmed by extensive fingerprinting approaches based on genes coding for polyketide synthases and nonribosomal peptide synthetases. The observed biosynthetic potential was correlated with the antibacterial activity exhibited by these isolates in laboratory conditions. Ultimately, the results demonstrate that intertidal sediment is a rich source of diverse cultivable Actinobacteria with high potential to synthesize novel bioactive compounds in their genomes.

## Introduction

Infectious diseases and multidrug resistance of clinically relevant pathogens are threatening global human healthcare systems^1^. The development of new antibiotics is imperative to fight bacterial infections^2, 3^. Natural products, especially microbial metabolites, are a fertile source of bioactive scaffolds and serve as the foundation for the development of several life-saving antibiotics^4^. Despite their undeniable significance, technological hitches in screening and discovery of novel compounds conveyed an interim decline in the attention to microbial natural products^5–8^. However, a noteworthy renaissance is being felt in the current century, with the ongoing exploration of microorganisms of underexplored sources, through the innovations in analytical and genome mining methods^5–7, 9^. Actinobacteria are the foremost runner in the production of biologically active scaffolds, especially anti-infectives^10, 11^. These commercially important Gram-positive bacteria have largely been isolated from both aquatic and terrestrial environments^12–15^. High-throughput genome sequencing methods expand our understanding of the biosynthetic potential of previously isolated Actinobacteria^16–18^, while metagenomes reveal the presence of many novel isolates that were previously undetected in cultivation studies^19^. Ultimately, isolation and exploitation of Actinobacteria from diverse environments are significant and could be continued for marine Actinobacteria^20–23^. Marine Actinobacteria evolved with unique physiological, chemical, and structural features that enable them to survive under the varying pressure, salinity, and temperature occurring in marine ecosystems^24–26^. In fact, such marine organisms are further endowed with the ability to produce novel molecules with interesting therapeutic applications not observed in their terrestrial counterparts, which is evident from various findings reporting the production of diverse bioactive compounds^20–27^.

Marine ecosystems have the potential to yield huge actinobacterial populations and their diversity is indisputable^22, 23^. Marine Actinobacteria are largely associated with marine sediments^28, 29^. This enormous diversity of marine Actinobacteria promises the discovery of bioactive molecules, and reflects growing international attention on marine microbial natural products. Holding true to this promise, many novel chemical structures have been discovered from marine Actinobacteria^21, 22, 30–35^.

Intertidal areas, which endure regular periods of immersion and emersion, are important in the coastal or estuarine environment and represent an underexplored biological niche that could be of interest for the discovery of novel biosynthetic genes and antimicrobials producing strains^36^. Diu (20.71°N 70.98°E) is a small island in the Arabian Sea near the Saurashtra Peninsula of Gujarat (India) covering an area of 38.8 km^2^. Intertidal regions of this island would be a potential source of unique and diverse microorganisms; however, there is no report on the isolation and characterization of Actinobacteria from this region. In the study presented here, a culture-dependent approach was undertaken to evaluate the biodiversity and biosynthetic potential of Actinobacteria from intertidal sediment samples. The results illustrate the high levels of diversity and biosynthetic potential dictated in terms of genomic fingerprints.

## Results

### Actinobacterial isolates

**Figure 1:**
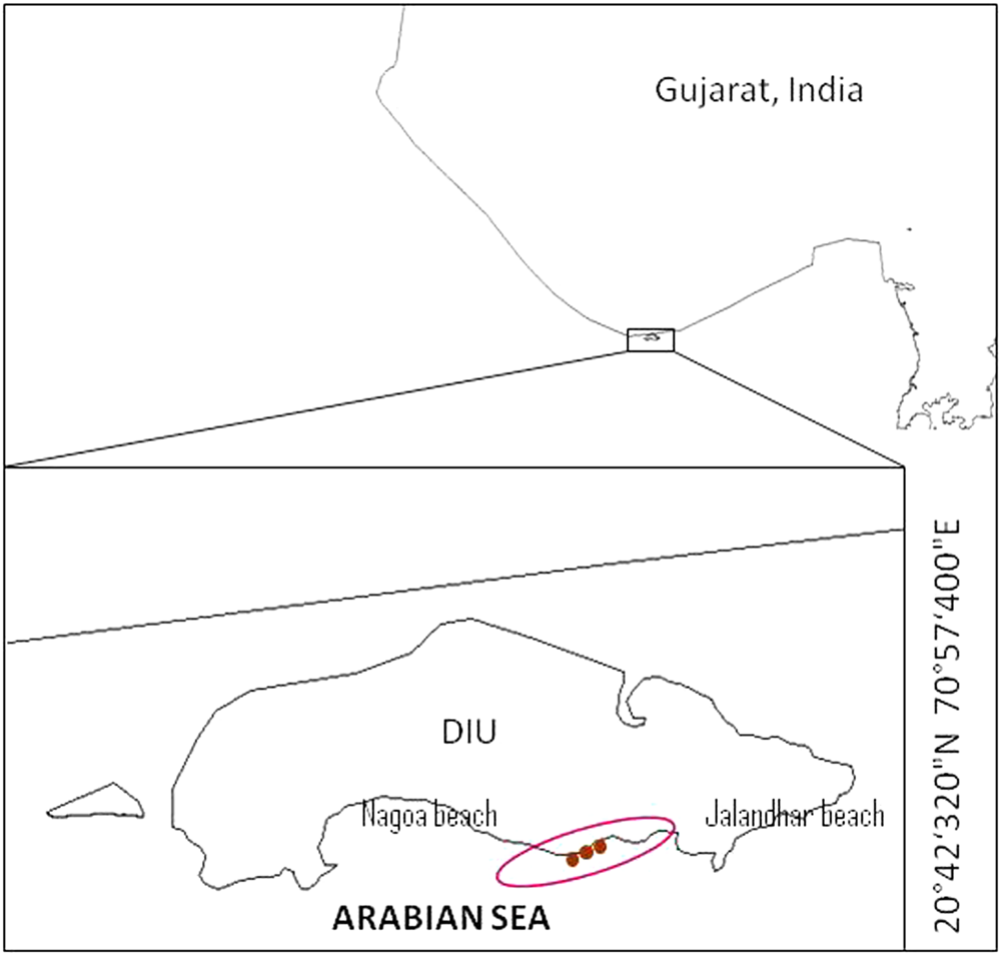
Sampling site map. Circle indicates sampling spots (20°42′320″N 70°57′400″E), intertidal region of Diu Island in the Arabian Sea. The map was generated using DIVA-GIS 7.5 (http://www.diva-gis.org/).

Eighteen sediment samples were collected from three different locations on Diu Island, Gujarat (Fig. 1), and used for selective isolation of Actinobacteria with eight different agar media. A total of 148 isolates with colony morphologies indicative of the order Actinomycetales by their characteristic aerial or substrate mycelium, and pigmentation, were identified. Most of the actinobacterial colonies were visible by the second week of incubation; however, some took as long as 5 weeks to appear on isolation agar. Among the isolates, 126 (85.1%) isolates formed well-defined aerial mycelium, while the remaining 22 (14.9%) lacked areal hyphae. Some isolates were initially orange in colour and turned dark after 20 days upon sporulation.

### Diversity and taxonomic profile

Out of 148 isolates, 66 representatives based on colony morphology, colour of aerial and substrate mycelia, and diffusible pigment production on M1 media were selected for genomic DNA isolation and sequencing of the 16S rRNA gene. The taxonomic diversity was evaluated based on the partial 16S rRNA gene sequences. Blastn analysis of 16S rRNA gene sequences against those in the NCBI GenBank database revealed phylogenetic affiliations to seven different families, Glycomycetaceae, Micromonosporaceae, Nocardiaceae, Nocardiopsaceae, Pseudonocardiaceae, Streptomycetaceae, and Thermomonosporaceae. At a genus level, the isolates were found to be affiliated with *Streptomyces*, *Micromonospora*, *Nocardiopsis*, *Saccharomonospora*, *Actinomadura*, *Glycomyces*, and *Nocardia*.

In the calculation of operational taxonomic units (OTUs), the isolates were clustered on sequence identities of 100%, 99%, and 98%. Four isolates were identified as duplicates using 100% sequence identity, where 66 isolates clustered into 62 OTUs. OTUs calculated based on sequence identities of 99% and 98% revealed 39 and 25 OTUs, respectively. At 99% sequence identity, *Streptomyces* accounted for 61.5% of OTUs, followed by *Micromonospora* (15.4%), *Nocardiopsis* (10.2%), *Saccharomonospora* (5.1%), *Actinomadura* (2.6%), *Glycomyces* (2.6%), and *Nocardia* (2.6%). Overall, 39 OTUs assigned to seven different genera showed ≤99% 16S rRNA gene sequence similarity with validly published type strains of species of *Streptomyces*, *Micromonospora*, *Nocardiopsis*, *Actinomadura*, *Glycomyces*, and *Nocardia*.

**Figure 2:**
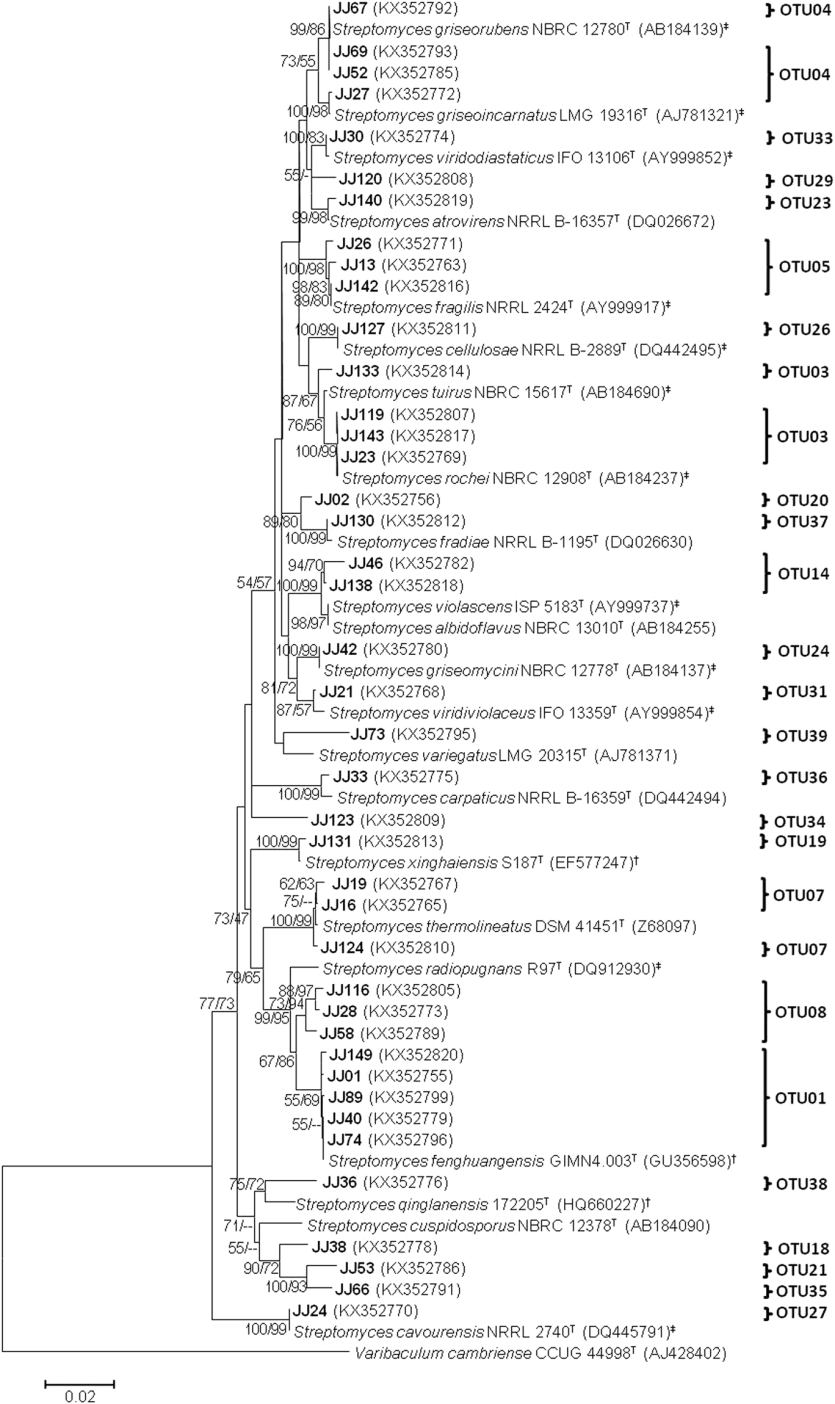
Maximum-likelihood (ML) phylogenetic tree of the *Streptomyces* based on 16S rRNA gene sequences of representative isolates of this study and nearest type strains. Isolates of this study are shown in bold font. GenBank accession numbers of 16S rRNA sequences are given in parentheses. ^#^Type strains with terrestrial origin. ^†^Type strains with marine origin. Bootstrap values (>50%) of the NJ and ML analyses are shown above the internodes before and after the backslash, respectively. Scale bar corresponds to 0.02 substitutions per nucleotide position.

Phylogenetic relationships of the representative isolates affiliated to *Streptomyces* and rare Actinobacteria (non-streptomycetes) were analysed by both neighbour-joining (NJ) and maximum-likelihood (ML) methods. Forty-one isolates of 24 OTUs were affiliated to *Streptomyces* (Fig. 2). These isolates shared 97.9% to 100% 16 S rRNA gene sequence similarity with species of the genus *Streptomyces*. Six isolates, JJ36 (OTU38), JJ38 (OTU18), JJ53 (OTU21), JJ66 (OTU35), JJ73 (OTU39), and JJ123 (OTU34) shared 97.6% to 98.6% sequence similarity and were loosely related to the nearest relatives. Isolates JJ73 and JJ123 found unique positions with their highest 16S rRNA gene sequence similarity, 97.9% and 98.6%, respectively, to species of *Streptomyces*. Four isolates JJ36, JJ38, JJ53, and JJ66 shared 97.6% to 98.5% sequence similarity and formed a separate clade along with two type strains *Streptomyces cuspidosporus* NRRL 12378^T^ and *Streptomyces qinglanensis* 172205^T^. Interestingly, the isolates that showed a phylogenetic relationship with the type strains of terrestrial origin occupied the upper half of the phylogenetic tree (Fig. 2), while the lower half was mainly dominated by streptomycetes of marine origin. Among the *Streptomyces* of this study, 29 (70.7%) isolates showed terrestrial origin, and the remaining 12 (29.3%) isolates showed marine origin.

**Figure 3:**
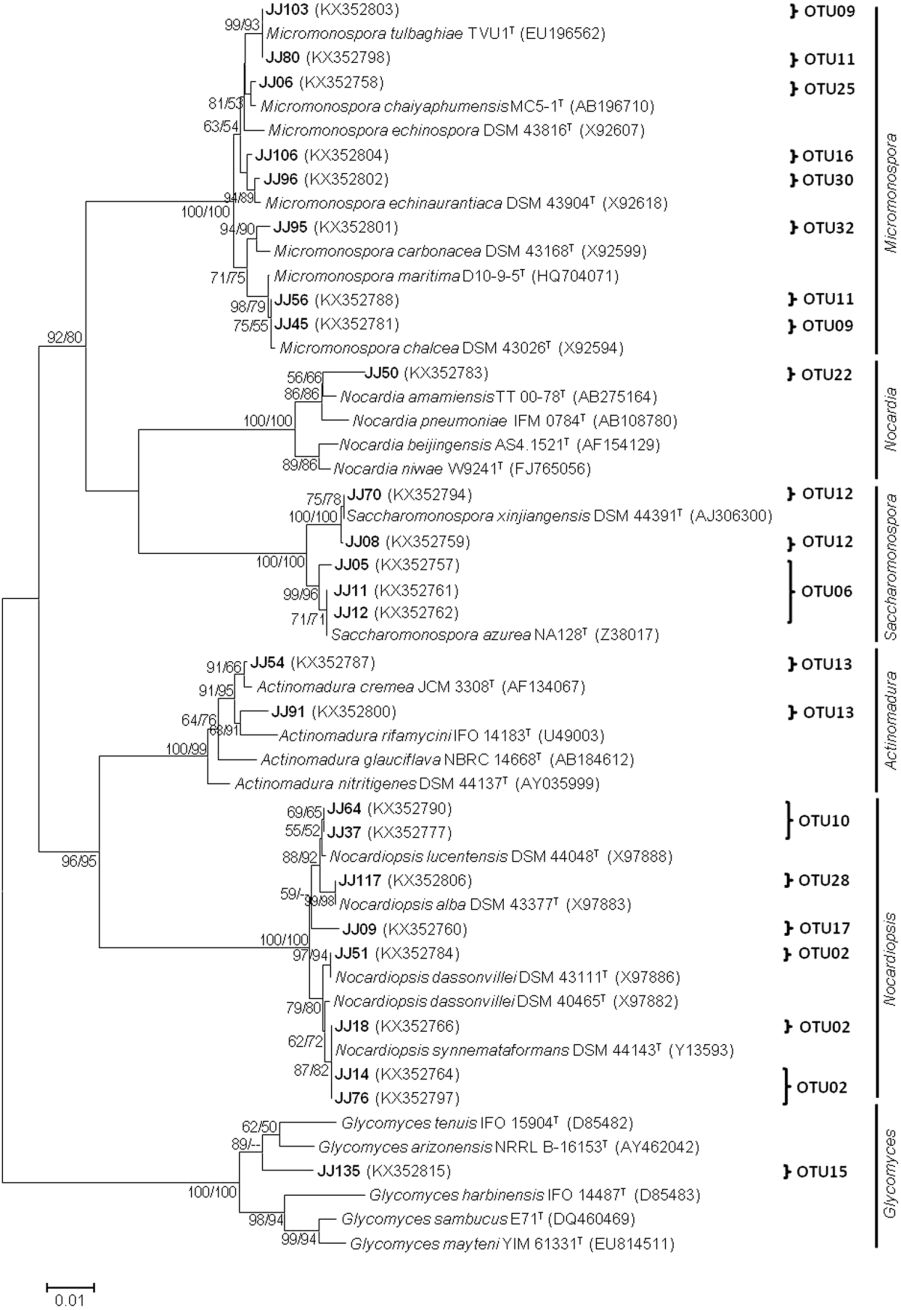
Maximum-likelihood phylogenetic tree of the *Micromonospora*, *Nocardiopsis*, *Saccharomonospora*, *Actinomadura*, *Glycomyces*, and *Nocardia* based on 16S rRNA gene sequences of representative isolates and nearest type strains. Isolates of this study are shown in bold font. GenBank accession numbers of 16S rRNA sequences are given in parentheses. Bootstrap values (>50%) of the neighbor-joining (NJ) and maximum-likelihood (ML) analyses are shown above the internodes before and after the backslash, respectively. Scale bar corresponds to 0.01 substitutions per nucleotide position.

The phylogenetic positions of 25 isolates belonging to 15 OTUs were assigned to at least 19 phylogenetic lineages within six genera, *Micromonospora*, *Nocardiopsis*, *Saccharomonospora*, *Actinomadura*, *Glycomyces*, and *Nocardia* (Fig. 3). The eight isolates belonging to *Micromonospora* shared 98.9% to 99.8% 16S rRNA gene sequence similarity with the nearest type strains. Isolate JJ95 (OTU32) showed lower sequence similarity (98.9%) with the nearest type strain, *Micromonospora carbonacea* DSM 43168^T^. Isolates JJ106 (OTU16) and JJ96 (OTU30) shared a single type strain *Micromonospora echinaurantiaca* DSM 43904^T^ with 99.2% and 99.8% sequence similarity, respectively. OTUs 09 and 11 contained two isolates each. It is noteworthy that one isolate from each OTU showed a different phylogenetic position. Isolates JJ103 of OTU09, as well as JJ80 of OTU11, showed a phylogenetic relationship to *Micromonospora tulbaghiae* TUV1^T^. Isolate JJ50 was the only isolate affiliated to *Nocardia* in this study. It shared 98.5% sequence similarity with *Nocardia amamiensis* TT 00-78^T^. Five isolates belonging to two OTUs of *Saccharomonospora* within the family Pseudonocardiaceae were assigned to at least three phylogenetic lineages. OTU13 represents the genus *Actinomadura* and comprised two isolates, JJ54 and JJ91. Isolate JJ54 showed 99.6% sequence similarity to *Actinomadura cremea* JCM 3308^T^, while JJ91 showed lower sequence similarity (98.7%) to *Actinomadura rifamycini* IFO 14183^T^. In the case of *Nocardiopsis*, eight isolates (JJ64, JJ37, JJ117, JJ09, JJ51, JJ18, JJ14, and JJ76) contained in four OTUs shared 98.6% to 99.9% 16S rRNA gene sequence similarity and at least five phylogenetic lineages. Among these isolates, Isolate JJ09 showed 98.6% sequence similarity and formed a stable monophyletic line that was shown to separate the isolates within the genus *Nocardiopsis*. Within the genus *Glycomyces*, JJ135 shared the highest similarity (97.6%) to the 16S rRNA gene sequence of type strain *Glycomyces arizonensis* (NRRL B-16153^T^ and formed a distinct branch.

### Screening for biosynthetic potential

**Figure 4:**
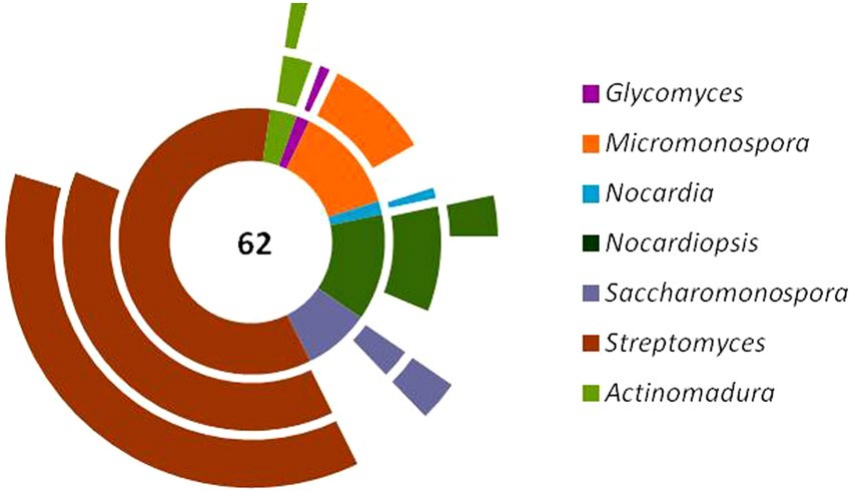
Doughnut chart summarising the proposition of NRPS and PKS-II positive isolates among the distinct actinobacterial isolates affiliated to different genus. The two levels of ripples around the doughnut represent the percentage of Actinobacteria that were positive for NRPS (inner ripple) and PKS-II (outer ripple). The number in the centre of doughnut indicates total actinobacterial isolates tested.

In the PCR based screening for biosynthetic potential, presence of non-ribosomal peptides synthetase (NRPS) and polyketide synthase type II (PKS-II) systems was checked by targeting adenylation and ketosynthase alpha domains, respectively. The screening was carried out in 62 distinct actinobacterial isolates (after omitting 4 duplicate isolates having 100% 16S rRNA gene sequence homology) to ascertain their biosynthetic potential. The presence of these genes was considered positive whenever a strong unambiguous amplicon of the right size could be observed. The observed numbers of NRPS and PKS-II positive isolates affiliated to different genus are summarized in Table S1. A total of 50 (80.6%) isolates were found to be positive for either or both NRPS and PKS-II. Among them, streptomycetes and rare Actinobacteria contributed 46.8% (29 isolates) and 33.9% (21 isolates), respectively. NRPS was predominant and found in 67.7% of Actinobacteria compared to 45.1% that were positive for PKS-II. This predominance was contributed by positive isolates that affiliated to all the genera *Glycomyces*, *Micromonospora*, *Nocardia*, *Nocardiopsis*, *Saccharomonospora*, *Streptomyces*, and *Actinomadura* (Fig. 4). PKS-II was found in four genera, *Nocardiopsis*, *Saccharomonospora*, *Streptomyces*, and *Actinomadura*, but not in *Glycomyces*, *Micromonospora*, and *Nocardia*. Twelve isolates collectively from four genera were not positive for both the PKS-II and NRPS. However, none of the seven genera was entirely negative for both PKS-II and NRPS.

### Screening for antibacterial activity

**Figure 5:**
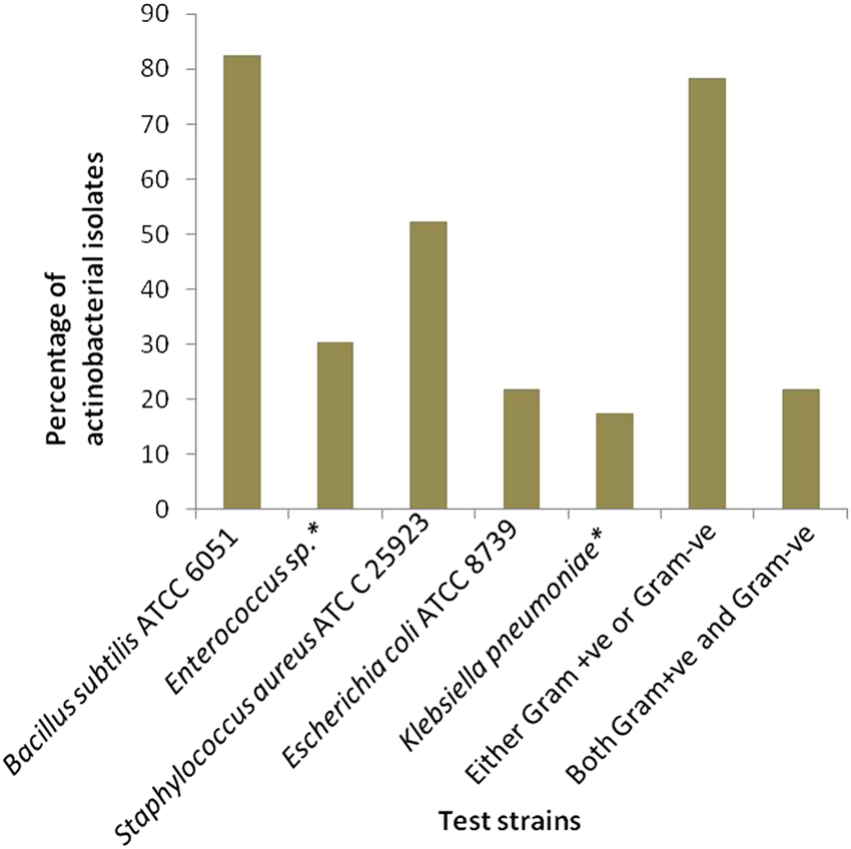
Bar chart summarising percentage of active actinobacterial isolates against different test strains (clinical isolates marked with ‘*’).

Sixty-two distinct actinobacterial isolates were further screened for antibacterial activities against 2 multi-drug resistant clinical isolates (*Enterococcus* sp. and *Klebsiella pneumonia*) and 3 type cultures (*Bacillus subtilis* ATCC 6051, *Staphylococcus aureus* ATCC 25923 and *Escherichia coli* ATCC 8739). Antibiotic-resistance profile of these 5 test strains was assessed and data are summarised in Table S2. Out of 62 isolates, 23 were positive against one or more test bacterial strains. The positive isolates belonged to different genera, *Streptomyces* (19), *Noardiopsis* (2), *Micromonospora* (1) and *Saccharomonospora* (1). Majority (78.3%) of the positive isolates showed inhibitory activity against Gram- positive bacteria (Fig. 5). Notably, eight isolates were active against vancomycin-resistant *Enterococcus* sp. Seven isolates were found to be active against Gram-negative bacteria, of these 4 were active against multi-drug resistant *Klebsiella pneumoniae*.

**Figure 6:**
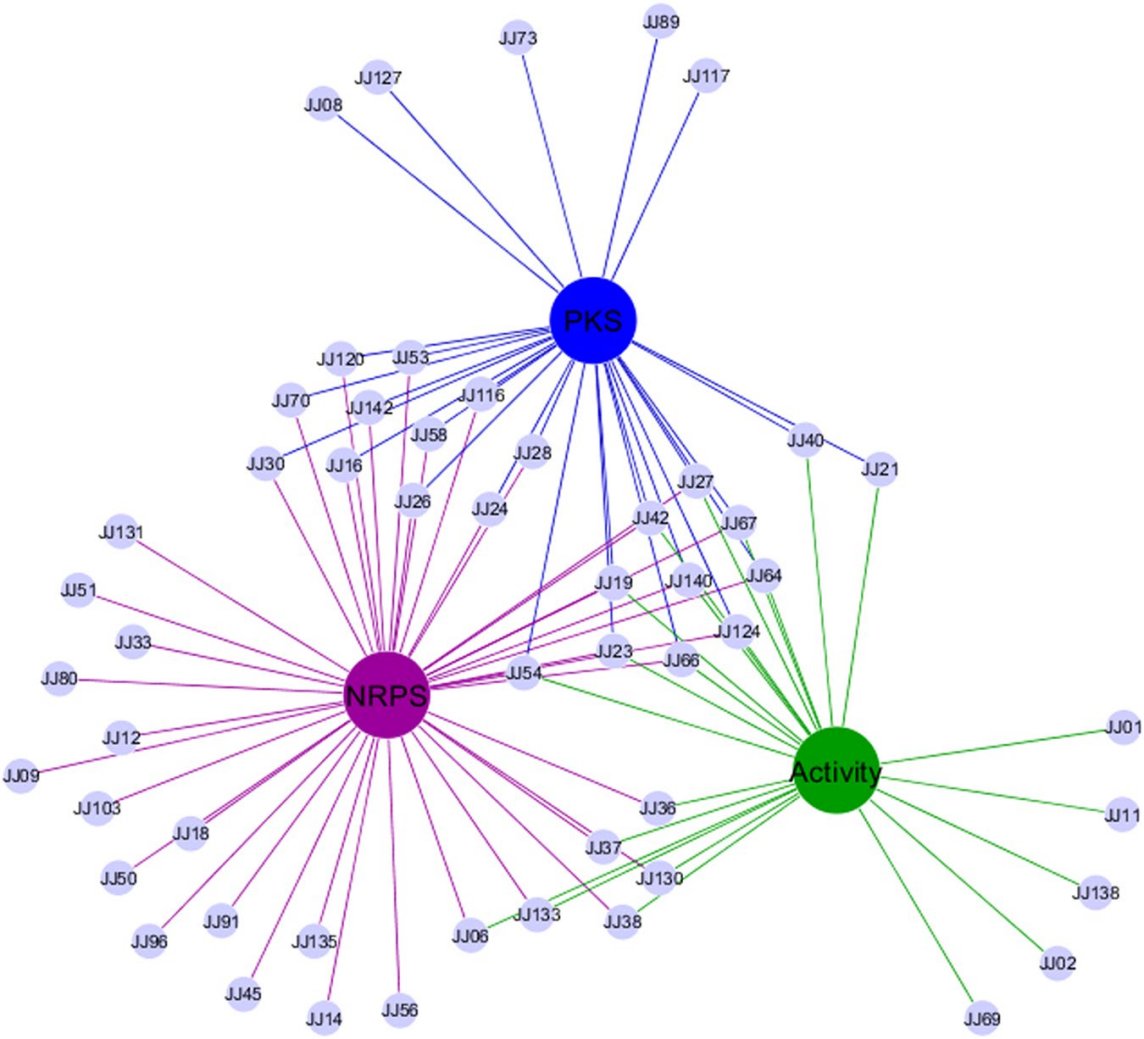
Correlation of antibacterial activity and occurrence of NRPS and PKS-II systems visualized by cytoscape network image.

The occurrence of NRPS and PKS-II was correlated with the detected antimicrobial activity of the isolates, and the relationship was visualized in a Cytoscape network image (Fig. 6). Among the 23 actinobacterial isolates that showed antibacterial activity, ten (43.4%) were positive for both PKS-II and NRPS. Eight isolates (34.8%) were positive for either PKS-II or NRPS while remaining five (21.8%) were negative for either. The *Streptomyces* constituted a majority (19, 82.6%) of antibacterial isolates, and most of them (15) were positive for either NRPS, PKS-II or both. Additionally, a significant number of actinobacterial isolates (31) showed no antibacterial activity while bearing either NRPS, PKS-II or both.

### Amplified fragment restriction analysis

The diversity of NRPS and PKS-II that indicates the biotechnological potential of the isolates was examined using a fingerprinting approach, based on restriction analysis of their amplified gene fragments. A combination of two widely used restriction enzymes with 4-bp recognition sites (*Alu*I and *Hae*III)^37^ was used to generate the restriction fingerprints.

To validate the suitability of individual restriction enzymes, *Alu*I and *Hae*III were independently used for generating restriction fingerprints of amplified NRPS and PKS-II fragments. *Alu*I generated moderately complex fingerprints, which consisted of 29 and 17 distinct restriction fragments for NRPS and PKS-II, respectively. In the subsequent UPGMA cluster analysis, 42 NRPS positive isolates were grouped under 33 groups (Figure S1), whereas 28 PKS-II positives were grouped under 12 groups at similarity coefficient value 0.93 (Figure S2). In the case of *Hae*III, restriction fragments were found to be more complex, scoring 38 and 24 distinct fragments for NRPS and PKS-II, respectively. The isolates were allocated to 37 and 18 groups in a UPGMA dendrogram based on NRPS (Figure S3) and PKS-II (Figure S4) fingerprints, respectively. The similarity coefficient value was set at 0.93 for the above grouping.

**Figure 7:**
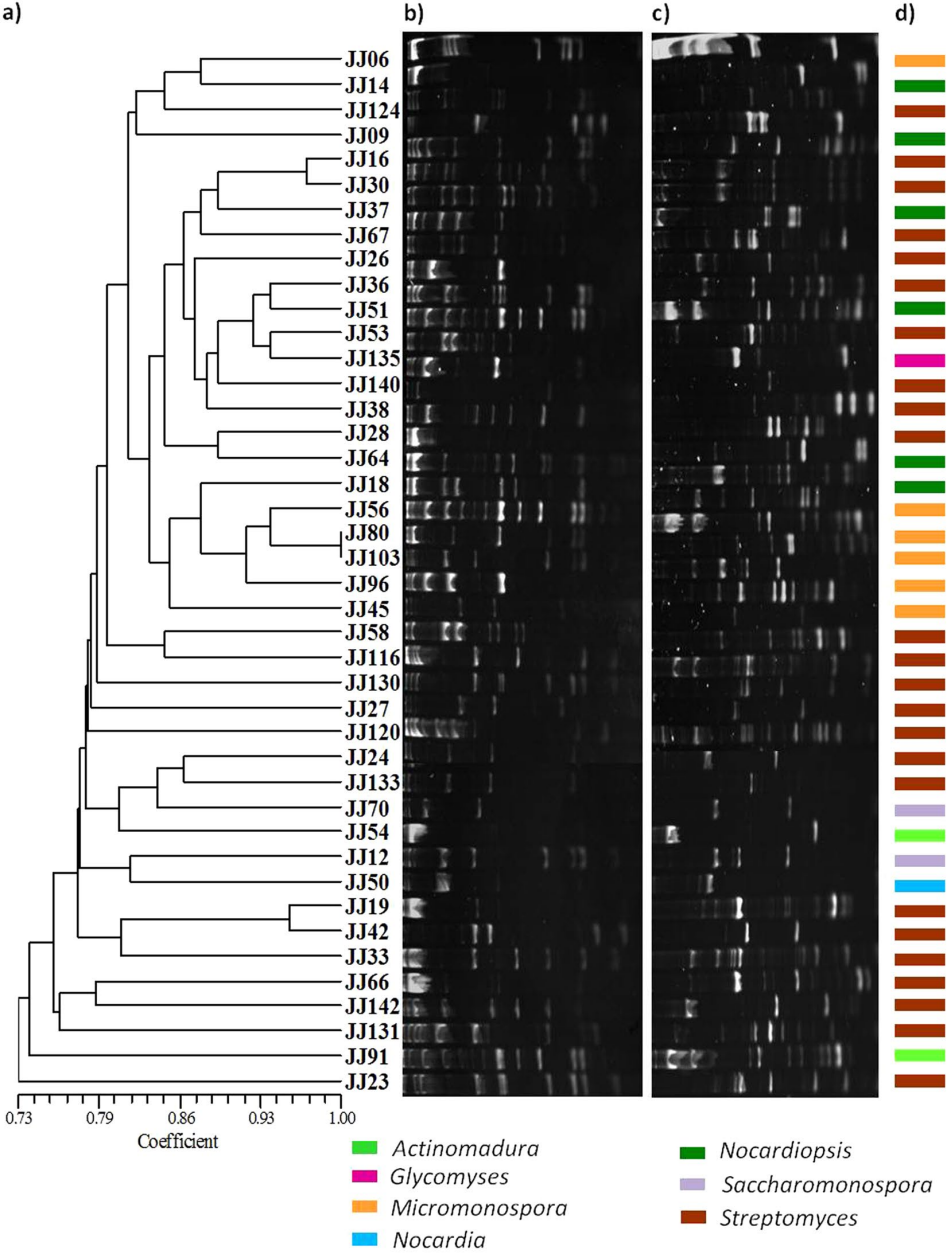
Diversity of NRPS studied by amplified fragment restriction fingerprinting method. UPGMA dendrogram (**a**) was inferred by restriction pattern of NRPS amplicons digested by *Hae*III (**b**) and *Alu*I (**c**). Lanes in the gels are in original order; irrespective of isolates appear in dendrogram. The gels were analyzed by densitometry and the bands whose areas were greater than 5% of the whole lane area were used for generating binary matrix. The similarities were calculated using the Jaccard’s coefficient, and the clustering was done by using the unweighted pair group method. Genus level affiliation of the isolates is also shown (**d**). Original gels are presented in supplementary Figure S5.

**Figure 8:**
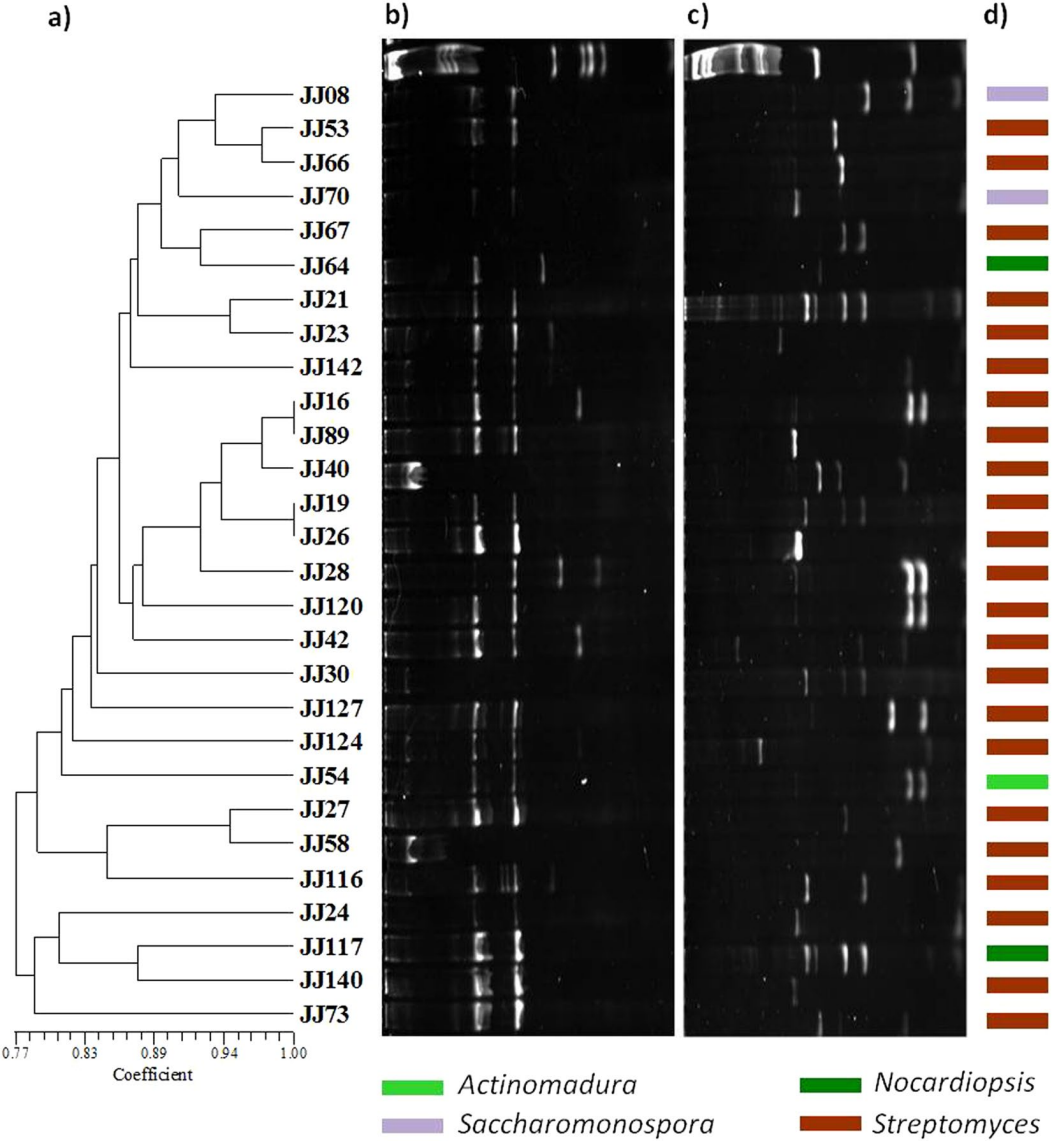
Diversity of PKS-II studied by amplified fragment restriction fingerprinting method. UPGMA dendrogram (**a**) was inferred by restriction pattern of PKS-II amplicons digested by *Hae*III (**b**) and *Alu*I (**c**). Lanes in the gels are in original order; irrespective of isolates appear in dendrogram. The gels were analyzed by densitometry and the bands whose areas were greater than 5% of the whole lane area were used for generating binary matrix. The similarities were calculated using the Jaccard’s coefficient, and the clustering was done by using the unweighted pair group method. Genus level affiliation of the isolates is also shown (**d**). Original gels are presented in supplementary Figure S6.

Restriction fingerprints generated by both *Alu*I and *Hae*III were used to construct a UPGMA dendrogram, and potential relatedness among the actinobacterial isolates was defined. Isolates presented distinct NRPS (Fig. 7) and PKS-II (Fig. 8) restriction patterns, irrespective of their ribosomal fingerprint patterns. High pattern heterogeneity was observed among the isolates classified within *Streptomyces* and *Nocardiopsis*. In contrast, high similarity of restriction patterns was observed between unrelated isolates of *Micromonospora*. Restriction fingerprints among the rare genera such as *Nocardia* and *Actinomadura* were very variable and, probably, not representative due to the small number of isolates.

### Analysis of biosynthetic gene sequences

Correctly sized inserts from the NRPS (12) and PKS-II (14) clones of seven strains were sequenced and subjected to detailed sequence analysis in an effort to assess diversity and biosynthetic richness. In the Blastx analysis against the nr protein sequence database (NCBI), the top matches were for adenylation domain of NRPS or the alpha domain of the PKS-II, confirming that the correct loci had been amplified. The Blastx results, closest match, E-value, and percentage similarity of 11 unique inserts that specify NRPS or PKS-II are summarized in Table S3.

Overall, NRPS sequence identities to the closest hits from the Blastx analysis ranged from 73% to 99%, and belonged to the phylum Actinobacteria. Despite strain JJ23 showing a very high amino acid sequence similarity of 99% to a domain of peptide synthetase from *Streptomyces* sp. SCAU5027, other strains harboured amino acid sequences with maximal identities of only up to 90%. Interestingly, strain JJ06 harboured two distinct NRPSs, one showing identity to *Streptomyces* sp. CNS606 (82%), the other to a gentamicin producer, *Micromonospora pallida* DSM 43817 (90%). The obtained amino acid sequences aligned partially to conserved domains of annotated NRPS sequences.

Translated PKS-II sequence of the tested strains showed similarity up to 99% with the previously described ketosynthase domains of PKS-II in *Streptomyces* strains. However, none of the sequences showed similarity to PKS-II of already-reported antibiotic producing strains. PKS-II of strains JJ23, JJ24, and JJ54 showed similarity to *Streptomyces* isolated from liquorice. Strain JJ40 harboured a ketosynthase protein domain of PKS-II with maximal identity (98%) to *Streptomyces* isolated from Indian coastal solar salterns. Strain JJ142 showed 91% homology to a putative type II PKS-ketosynthase alpha subunit of uncultured bacteria.

**Figure 9:**
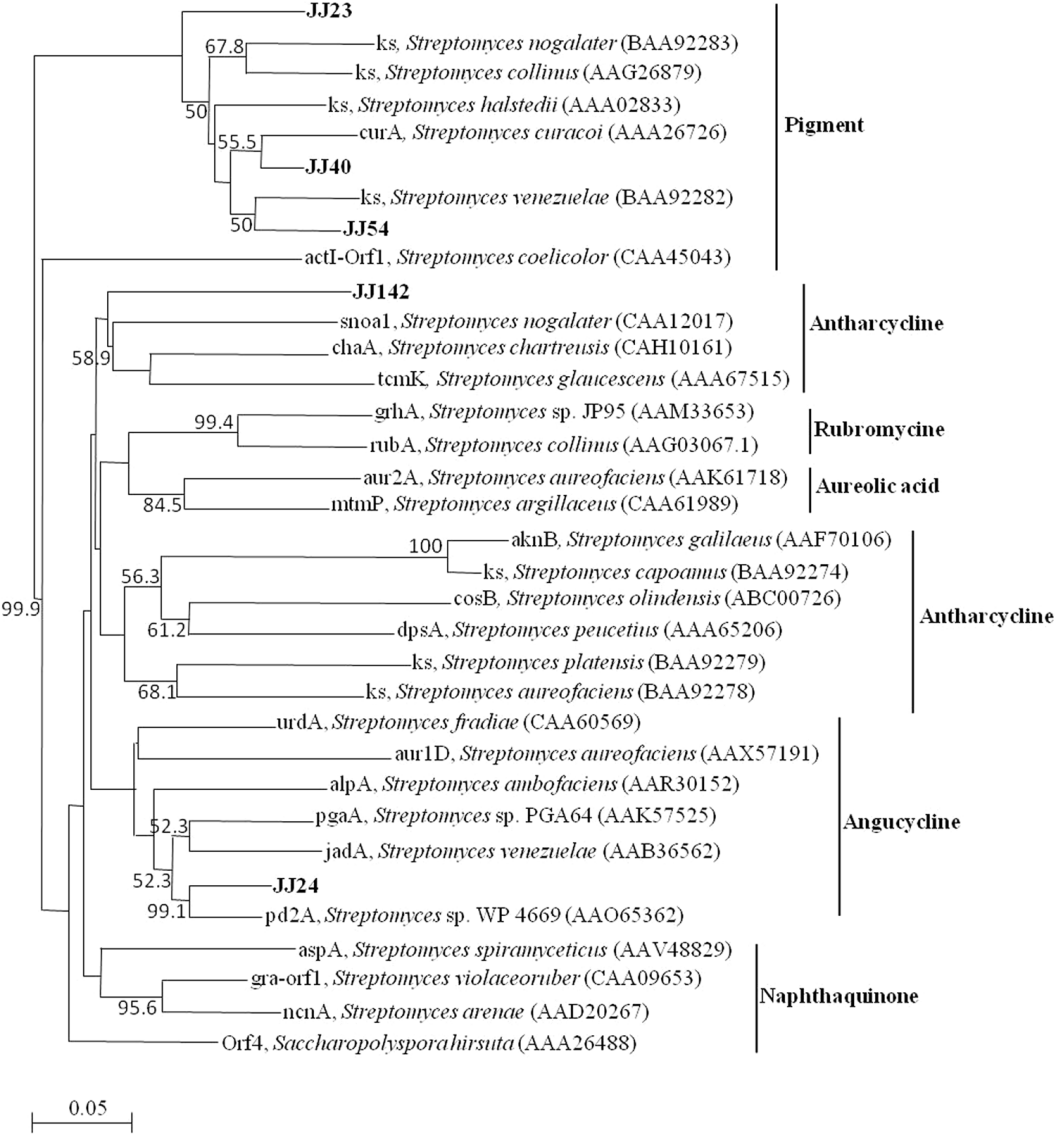
Neighbor-joining tree of PKS-II amino acid sequences of five strains (shown in bold) with reference sequences. Predicted chemical classes are indicated on the right. The scale bar indicates the number of substitutions that occur per site.

Phylogenetic analysis of amino acid sequences deduced from five PKS-II fragments of this study, with reference sequences, was done and the NJ tree is presented in Fig. 9. Significant diversity was found among the five PKS-II fragments that were distributed throughout various clades corresponding to different classes of compounds. Based on the heuristic rule that PKSs for the synthesis of structurally similar antibiotics share a high degree of sequence similarity, and the simplest assumption that PKS-II sequences in the same sub-cluster exhibit similar functions^38^, the biosynthetic potential of the five strains was predicted. Strains JJ23, JJ40, and JJ54 were distributed in the clade which covers pigment compounds; notably, the former one formed a very clear branch. JJ40 and JJ54 formed a branch with *S. curacoi* and *S. venezuelae*, respectively. Interestingly, JJ142 fell into the anthracycline-producing group with significant novelty. Two clones of JJ24 shared high-level similarity to each other and fell within the angucycline group with *Streptomyces* sp. WP 4699.

### Fermentation and secondary metabolites analysis

Secondary metabolites analysis of three isolates that showed antibacterial activity and biosynthetic potential was done using LC-MS-Q-TOF. A total of 23 different compounds were found in the crude extract from the spent broth of three strains JJ21, JJ23 and JJ42 (Table 1).

**Table 1:** Summary of putative novel and known compound detected by LC-MS-Q-TOF based secondary metabolites analysis performed in three isolates.

Strains	MS (*m/z*)	Known or putative novel secondary metabolites produced	Chemical/Structural class
JJ21	[M-H]^−^ = 335.40	Antibiotic SF2768^*^	Isonitrile-bearing antibiotic
	[M-H]^−^ = 563.79	Geldanamycin	Macrocyclic polyketide
	[M-H]^−^ = 311.50	1 putative novel compound	—
	[M-H]^−^ = 339.52	3 Octalactin B like compounds	Octalactin
JJ23	[M-H]^−^ = 269.56	10-Methylhexadecanoic acid derivative	—
	[M-H]^−^ = 325.70	1 putative novel compound	—
	[M-H]^−^ = 339.74	3 putative novel compounds	—
	[M-H]^−^ = v564.15	3 putative novel compounds	—
JJ42	[M-H]^−^ = 560.11	1 putative novel compound	—
	[M-H]^−^ = 213.41	4 Anmindenol derivatives	Sesquiterpenoids
	[M-H]^−^ = 227.48	2 putative novel compounds	—
	[M-H]^−^ = 338.69	1 putative novel compound	—
	[M-H]^−^ = 241.53	1 putative novel compound	—

*Compounds with reports of antibacterial activity.

In a subsequent compounds purification study, two antibacterial compounds were isolated from the culture broth of *Streptomyces* sp. JJ21and designated as OBLC-02 and OBLC-03. Both these compounds were partially confirmed as octalactin-like compounds by comparison of their^1^H NMR data with that of reported octalactin^39^. The ^1^H NMR spectrum (supplementary data, Figure S7) of OBLC-02 showed the presence of an olefinic proton (δ_H_ 5.80–5.78), an oxymethine proton (δ_H_ 4.62–4.60) attached to alkyl substituted carbon in cyclic ring and five methyl protons (δ_H_ 2.10 - 0.91) that correlated well with that of octalactin. In comparison, OBLC-03 showed less number of methyl protons.

## Discussion

Marine Actinobacteria have remarkable ability to produce a wealth of metabolites with substantial structural complexity and exceptional biological activity. Our data elaborate the taxonomic diversity, phylogenetic novelty, and biosynthetic potential of cultivable Actinobacteria from intertidal marine sediments of Diu Island (India) in the Arabian Sea.

In this study, we performed a stamping method with eight different isolation media that favoured isolation of 148 actinobacterial isolates. These cultivable isolates belong to seven different genera, *Streptomyces*, *Micromonospora*, *Saccharomonospora*, *Nocardia*, *Nocardiopsis*, *Actinomadura*, and *Glycomyces*. There is no previous report on bioprospection of cultivable Actinobacteria from the intertidal sediments of Diu Island in the Arabian Sea, although Kokare *et al*.^40^, isolated 80 Actinobacteria from Alibag, Janjira, and Goa coastlines and assigned them to six genera. In this study, a higher abundance of Actinobacteria in Diu sediments was observed compared to other parts of the western coastline where Actinobacteria belonging to a maximum of five genera were isolated, using almost the same sample processing and cultivation conditions^40^. Besides the geographic deviation, seasonal variations with respect to temperature, tide, and other abiotic conditions might have affected the relative occurrence of Actinobacteria in this study, as sample collection was performed at different time of the year (early winter for this study and monsoon for previous study^40^).

The predominance of *Streptomyces* was observed in the sediment samples, as is also common in other reports^40, 41^. However, in the present study, members of six non-streptomycete genera constituting 40% of isolates were obtained, of which *Saccharomonospora*, *Actinomadura*, and *Glycomyces* were not observed in previous studies^40, 41^. The relative occurrence of non-streptomycetes and their significance have recently come to light^11, 42–44^, and reports have increased in recent decades^45, 46^. However, several genera including *Microbispora*, *Saccharomonospora*, and *Verrucosispora* are rarely observed in culture-dependent studies, and remain as rare Actinobacteria.

Based on 16S rRNA gene sequence identity at different thresholds, isolates were clustered into OTUs. The 100% threshold eliminated four isolates as duplicates from the group of isolates. According to the revised 16S rRNA gene sequence similarity threshold which is currently 99% for Actinobacteria^47^, representative isolates were clustered into 39 OTUs. In recent studies, 37 and 147 OTUs have been reported from 136 and 358 representative isolates, respectively from terrestrial^14^ and marine ecosystems^48^. Relatively high number of OTUs deduced from 66 representative isolates indicates the presence of many novel taxonomical groups. Using this metric, the lineages within these OTUs that exhibited ≤99% sequence similarity with validly reported species were considered to represent putative new species. Such putative new species formed distinct phyletic lines in the phylogenetic trees (Figs 2 and 3), supported by valid bootstrap values. Notably, novel isolates belong to the genera *Actinomadura*, *Glycomyces*, *Nocardiopsis*, *Nocardia*, and *Streptomyces*, and support further taxonomic studies of Actinobacteria from Diu. These results indicate that Diu intertidal sediments harbour readily cultivable novel Actinobacteria which can be explored for bioactive natural products.

Moreover, Blastn analysis against 16S rRNA gene sequences in the NCBI database suggests that the Actinobacteria inhabiting the intertidal sediments have either terrestrial or marine origin. In particular, a larger number of *Streptomyces* showed adaptation to the marine environment rather than originating from these environments. This observation suggested that intertidal regions are sites where terrestrial and marine Actinobacteria meet one another, and evolve under emersion stress. It was attested by phylogenetic trees (Fig. 2) in which *Streptomyces* of terrestrial origin occupied the upper half, while the lower half was mainly dominated by the strains of marine origin.

Underexplored environments continue to provide diverse Actinobacteria with novel biosynthetic potential. The biosynthetic potential of Actinobacteria could not be determined using bioactivity screening in limited production conditions. Genetic screening is an alternative approach which recognizes biosynthetic potential that could be systematically exploited for drug discovery^45, 48–50^. To assess the biosynthetic potential of the Actinobacteria, we adopted PCR-based screening, restriction fingerprinting, and phylogenetic analysis of functional gene fragments.

In the present study, genetic screening was done by targeting specific domains of PKS-II and NRPS that provide a rapid measure of biosynthetic potential and functional diversity within the isolates. This is because PKS (type I or type II), NRPS, and even PKS–NRPS hybrid pathways are responsible for most natural products with interesting biological activity^51, 52^. The PKS-II and NRPS primers used in this study successfully detected the targeted portion of these genes in a total of 50 (81%) actinobacterial isolates. This PCR-based screening revealed that most of the isolates bear biosynthetic genes coding for either or both PKS-II and NRPS that are widely distributed among the Actinobacteria^25^. Notably, the relative occurrence of NRPS was found in both *Streptomyces* and non-*Streptomyces*, which indicates a wider range of Actinobacteria of interest for bioprospecting. The relative presence of NRPS sequences in members of the genus *Micromonospora* is in agreement with previous results obtained in different isolates of the same genus^53, 54^. The occurrence of the PKS-II was observed in almost all the streptomycetes (96%), which furthers their already-recognized potential in secondary metabolism. Half of the non-*Streptomyces* belonging to *Saccharomonospora*, *Nocardiopsis*, and *Actinomadura* also showed evidence of PKS-II. The overall biosynthetic potential of the Diu-based isolates was either higher than or comparable to that of recent reports^45–50^. On these grounds, the different Actinobacteria cultured from the Diu sediments provide opportunities to explore these isolates for the genetic potential to produce secondary metabolites.

The diversity of NRPS and PKS-II among the isolates was examined using a fingerprinting approach based on restriction analysis of the amplified biosynthetic sequences. In the present study, two restriction enzymes *Hae*III and *Alu*I were used for the fingerprinting of PKS-II and NRPS. The results revealed that the availing restriction data generated with both enzymes are more valid and preferable to using a single enzyme^53^. Interestingly, a pattern of heterogeneity was observed in restriction profiles among the isolates at inter- and intra-genus levels, irrespective of their ribosomal fingerprint patterns. Most of the *Streptomyces* showed extremely different restriction profiles and constituted clusters across the dendrogram. Indeed, those that had closer relation in the 16S rRNA gene-based phylogeny also showed significant heterogeneity, which suggested a quite diverse biosynthetic potential even among closely related isolates. Similarly, isolates classified under *Nocardiopsis* and *Saccharomonospora* showed significant diversity, and correlates with the heterogeneity of PKS-II and NRPS gene clusters in *Nocardia* strains varied substantially depending on species. Overall, the high levels of heterogeneity in the restriction fragment fingerprints underscore the biosynthetic diversity and richness among the Actinobacteria of Diu intertidal sediments.

As a token of interest, some of the randomly selected PKS-II and NRPS fragments were sequenced and extensively studied to understand the novelty of the secondary metabolites with which they are involved. Blastx results revealed that the obtained sequences were part of either the PKS-II or NRPS. The deduced amino acid sequence based on phylogenetic analysis of PKS-II fragments done with a reference sequence reported by Metsä-Ketelä *et al*.^55^ endorsed categorization of the PKS-II sequences into functional chemical classes (Fig. 7). The results revealed that PKS-II of isolate JJ24 grouped with the naphthoquinone class of molecules, which also demonstrates antibiotic and antitumor activity.

Interestingly, the PKS-II of strain JJ142 formed a well-separated lineage within the anthracycline group, suggesting its potential to produce novel compounds related to this class of bioactive compounds. Moreover, most of the Blastx hits of the PKS-II fragment of JJ142 were uncultured bacteria, which indicate the novelty of this strain. Additionally, strain JJ142 showed significant phylogenetic distance in its 16S rRNA gene sequence (less than 96% similarity) from anthracycline-producing species of *Streptomyces*. Another interesting thing is that strain JJ54 is a member of the genus *Actinomadura*, but showing PKS-II sequence similarity to *Streptomyces*. It reflects the occurrence of horizontal gene events within members of a given actinobacterial community of intertidal sediments, as previously reported by Sigmund *et al*.^56^.

Antibacterial activity screening of distinct isolates demonstrated that more than two-thirds of the isolated Actinobacteria produce antibacterial metabolites that are readily accessible for further bioprospection to novel leads. The bioactivity was found to be either narrow against Gram-negative or Gram-positive bacteria, or broad against both types. The secondary metabolites analysis of three antibacterial strains led to the detection several putative novel compounds and some known compounds. Notably strains JJ23 and JJ42 were found to produce more number of putative compounds. It suggests that the compounds responsible for antibacterial activity of these strains are novel and they could find biotechnological importance. Detection of previously reported antibacterial compounds in strain JJ21 correlates with their antibacterial activity.

Cytoscape visualization of correlation between the prevalence of antibacterial activity and biosynthetic genes (PKS-II and NRPS) revealed that the prevalence of biosynthetic systems is high among the isolated strains when compared to the antibacterial activity which is relatively low. The prevalence of PKS-II and NRPS systems suggests that these strains possess the genetic capacity to produce some secondary metabolites if cultivated under alternative conditions that have an impact on secondary metabolite production^57, 58^. The strains could also be exploited through the genome-guided route of bioprospection^59, 60^. Collectively, these data demonstrate that all the distinct Actinobacteria isolated in this study from intertidal sediment have the potential to produce secondary metabolites.

The LC-MS-Q-TOF analysis of EtOAc extracts of three potential strains JJ21, JJ23 and JJ42 revealed that these strains produce several known as well as putatively novel compounds under normal laboratory conditions. Subsequently, two antibacterial compounds were purified and partially confirmed as octalactin-like compounds based on^1^H NMR spectra. To our knowledge, there is no previous report of antibacterial activities associated with octalactins. Our present study indicates that the purified octalactin-like compounds have inhibitory activity against vancomycin-resistant *Enterococcus*. Further work is in progress for complete characterization and validation.

In summary, this study is the first comprehensive investigation into the cultivable biodiversity and biosynthetic potential of Actinobacteria from previously unreported Diu intertidal sediments. The relatively small number of intertidal sediments that we sampled from Diu Island in the Arabian Sea harbours a significant variety of actinobacterial genera such as *Streptomyces*, *Micromonospora*, *Saccharomonospora*, *Nocardia*, *Nocardiopsis*, *Actinomadura*, and *Glycomyces*, many of which merit novel species. Considering that these different Actinobacteria provide opportunities to explore their biosynthetic potential which could be subsequently exploited for the discovery of novel natural products, their NRPS and PKS-II were fingerprinted. Ultimately, it is clear that intertidal sediments are a rich source of diverse cultivable Actinobacteria with high biosynthetic potential in their genomes. Further studies are underway to discover promising natural products that may have value in research and drug discovery programmes.

## Methods

### Sediment collection

A total of 18 marine sediment samples were collected from three inter-tidal locations along the coast of the Diu, Gujarat (Fig. 1), in November 2015. The collected samples were transported intact at ambient temperature to the laboratory and immediately processed for isolation of Actinobacteria.

### Selective isolation

All the composite sediment samples were dried in a laminar flow hood for 16 h prior to the selective isolation of Actinobacteria. Once dried, the samples were ground and inoculated using the plate stamping technique^29^ on 8 different isolation media (M1 to M8); the compositions of these media are given in Table S4 in the supplemental material. All the isolation media were amended with cyclohexamide (100 µg/mL) and nalidixic acid (50 µg/mL) to reduce fungal and fast growing bacteria, while select for Actinobacteria. The inoculated plates were incubated at 28 °C and regularly observed up to 12 weeks for appearance of actinobacterial colonies. Colonies were purified on the same media, and purified isolates preserved at −80 °C in 15% (v/v) glycerol.

The pure isolates were primarily grouped based on the presence or absence of aerial mycelium. Within these categories, strains were further grouped according to colony morphology, colour, and diffusible pigment production. Representatives as well as some duplicates from each group were selected for 16S rRNA gene sequencing and phylogenetic analysis

### 16S rRNA gene amplification and sequencing

The selected strains were cultured in 20 mL of medium M1 and shaken at 180 rpm and 28 °C for 3 to 7 days. Cells were harvested by centrifugation and genomic DNA extracted with FastDNA SPIN Kit (MP Biomedicals, USA) using the manufacturer’s protocol. The 16S rRNA gene was amplified using 10 pM of universal primers 27 f (5′-AGAGTTTGATCCTGGCTCAG-3′ and 1492r (5′-GGTTACCTTGTTACGACTT-3′), in 50 μL reaction mixture contained 1X reaction Buffer (Sigma, USA), 200 µM of dNTPs, each primer, 5 to 25 ng of genomic DNA and 0.05 U of Taq DNA polymerase enzyme (Sigma, USA). The thermo-cycling was started with an initial denaturalization at 94 °C for 8 min followed by 31 cycles at 94 °C for 1 min, 58 °C for1 min and 72 °C for 1.5 min, followed by a final extension at 72 °C for 5 min. The PCR products were purified and sequenced with the same primers, using an ABI 3730XL sequencer. The 16S rRNA gene sequences were deposited in GenBank under the accession numbers, KX352755 to KX352820.

### Operational taxonomic units (OTUs)

The resultant forward and reverse electropherograms were checked manually, edited and assembled using the Chromas Lite (http://chromas-lite.software.informer.com/) and EMBOSS merger (http://www.bioinformatics.nl/cgi-bin/emboss/merger) online tools. The assembled contigs of about 1,300 nucleotides were aligned using RDP online aligner^61^ and grouped into operational taxonomic units (OTUs) using the online tool Clusterer (http://www.bugaco.com/mioritic/) based on 98, 99 and 100% sequence identity. Each singleton and cluster was numbered as an OTU.

### Phylogenetic analysis

Phylogenetic trees were constructed based on the quality checked 16S rRNA gene sequences of the isolates and a set of related reference sequences, with neighbor-joining^62^ and maximum-likelihood methods^63^ in MEGA 5.0 package^64^. The confidence of the tree topologies was assessed by 1,000 bootstrap replicates.

### Screening for antibacterial activity

Actinobacterial isolates were screened for antibacterial activity against panel of bacteria (Table S2) by agar-plug method. In agar plug method, the isolates were initially inoculated over M1 agar media (Table S4) in Petri dishes and incubated at 28 °C for 7 to 12 days. After the incubation, agar plugs of 6 mm in diameter were cut from the actinobacterial plates, and plugged into the wells bored (diameter of 6 mm) in Mueller Hinton (MH) agar plates seeded with test bacteria. The MH plates were incubated at 37 °C for 24 hours and observed for zone of inhibition around the inserted agar plugs.

### PCR amplification of PKS-II and NRPS fragments

Ketosynthase alpha domain of polyketide synthase type II (PKS-II) was amplified from genomic DNA of the representative isolates, using the degenerative primers set, PKS-F (5′-GGC AAC GCC TAC CAC ATG CAN GGN YT-3′) and PKS-R (5′-GGT CCG CGG GAC GTA RTC NAR RTC-3′)^65^. Similarity, adenylation domain of NRPS system was targeted using the degenerate primers set, NRPSF 5′- CGC GCG CAT GTA CTG GAC NGG NGA YYT -3′ and NRPSR (5′- GGA GTG GCC GCC CAR NYB RAA RAA -3′)^65^. Reaction mixture contained 200 µM of each dNTP, 0.5% DMSO (v/v), 1 *m*M of degenerate primers, 25 ng of genomic DNA and 0.5 units of Taq polymerase (Sigma-Aldrich, USA) in 50 µL of 1X PCR buffer. The PCR was performed in a BioRad T100 Thermal cycler (BioRad, USA), with the thermal cycling started with initial denaturation at 95 °C for 5 min, followed by a 30 cycles of denaturation at 95 °C for 30 Sec; annealing at either 61 °C (PKS-II) or 63 °C (NRPS) for 45 Sec and extension at 72 °C for 90 Sec, and a final extension at 72 °C for 10 min. *Streptomyces kanamyceticus* MTCC 324 and *Micromonospora echinospora* MTCC 930 were used as the positive controls with each set of reactions, while a negative control lacks template. All PCR products were electrophoresed along with GeneRuler DNA Ladder Mix (Fermentas, USA) on 1% agarose gel (w/v) in TBE buffer. The expected size of PCR amplicons was 350 bp and 480 bp for PKS-II and NRPS, respectively.

### Amplified fragment restriction and data analysis

All correctly sized PCR products were subjected to restriction profiling to detect polymorphism of PKS-II and NRPS harboured by the isolated Actinobacteria. The amplified PKS-II and NRPS fragments were independently digested by *Alu*I and *Hae*III (New England Biolabs, Inc.), following the protocol suggested by manufacturer. Digested fragments were separated on 10% of polyacrylamide gel (w/v) and visualized using ethidium-bromide. The bands whose area percentage higher than 5% of the whole lane area was scored and binary data were generated. Subsequently, the binary data processed to distance matrix and dendograms were drawn using unweighted pairgroup arithmetic mean algorithm^66^ (UPGMA) by NTSYSpc v.2.

### Cloning and sequencing of the amplified KS and NRPS fragments

The amplified PKS-II and NRPS fragments (PCR products) were purified using the High Pure PCR product purification kit (Roche Molecular, Basel, Switzerland), following the manufacturer’s protocols. The purified PCR products were cloned using pGEM-T Easy Vector System I (Promega) with DH5α *E.coli* cells, according to manufactorer’s protocols. Transformed clones were cultured in 3 mL Luria-Bertani broth with ampicillin to a final concentration of 100 µg/mL. Plasmids DNA was isolated using Pure-Link Quick Plasmid Miniprep Kit (Invitogen, Life Technologies), following the manufacturer’s protocols. PKS-II and NRPS inserts were sequenced using the M13F primer. Vector contaminations were removed from the resulting sequence using VecScreen online-tool (https://www.ncbi.nlm.nih.gov/tools/vecscreen/). The PKS-II and NRPS sequences were deposited in GenBank under the accession numbers, MF443243 to MF443247 and MF443237 to MF443242, respectively.

### Homology search and phylogenetic analysis

The obtained PKS-II and NRPS fragment sequences were analysed against public databases using Blastx online tool (http://www.ncbi.nlm.nih.gov/BLAST/). ORFs in the contigs were identified using ORF finder (https://www.ncbi.nlm.nih.gov/orffinder/). The deduced amino acid sequences were aligned with 29 reference sequences and a phylogenetic tree was constructed using neighbour-joining method in MEGA5. The representative PKS-II sequences have been described in earlier studies^43^.

### Fermentation and secondary metabolites analysis

Frozen stock of selected strains was inoculated into 250 mL Erlenmeyer flasks containing 50 mL M1 broth prepared with 70% seawater (v/v). These starter cultures were incubated at 28 °C on a rotary shaker (180 rpm) for 5 days. An 8 mL aliquot of the starter cultures was then transferred to 2 L Erlenmeyer flask containing 800 mL of same media, and incubated at 28 °C on a rotary shaker with 180 rpm for 10 days. Spent broth was harvested by centrifugation, and extracted with equal volume of ethyl acetate (EtOAc). The organic layer was separated and concentrated to dryness in a vacuum and suspended in 1.5 mL methanol. Ten microliters of extract were subjected to chromatographic separation over SUPELCO 516 C-18 column (5 µm, 250mm × 4.6 mm) on an Alliance Separations Module (Alliance 2690, Waters).The mobile phase consisted of water containing 0.1% formic acid (A) and methanol (B). The gradient condition was as follows: 0 to 10% B at 0.5 min, 40% B at 40 min, 55% B at 7 min, 80% B at 15 min, 100% B at 22 min and hold up to 30 min, and finally 0% B at 42 min. Column eluent was analyzed using a Micromass Q*-*TOF Mass Spectrometer (Micromass 2487, Waters) equipped with an electrospray ionization (ESI) source in negative ion mode. Instrument parameters were set as follows: capillary voltage, 2.5 kV; sample cone voltage, 25 V; source temperature, 110 °C; desolvation temperature, 200 °C; nitrogen as the collision gas. Data acquisition and processing were done by Mass Lynx Software version 4.1 (Waters, USA). The compounds were identified by the comparison of molecular weights, mass features and retention times with published chemical data from standard databases (Dictionary of Natural Products, version 29.2; SciFinder, 2017) and literature.

### Purification and characterization of bioactive compounds

The EtOAc extract of *Streptomyces* sp. JJ21 culture broth was partitioned on a preparative HPLC (LC-20AP, SPD-M20A, FRC-10A; SHIMADZU) using an Enable C18 column (250 × 20 mm, 10 μm; Sigma) with a gradient solvent system (10% to 100% MeOH in H_2_O over 64 min; flow rate, 9.45 ml/min). A total of 32 fractions with different retention time were collected and tested for antibacterial activity by disc diffusion method (data not shown), against vancomycin-resistant *Enterococcus* species. Active fractions were concentrated under reduced pressure at room temperature and used for structural characterization. The^1^H NMR spectra at 600 MHz were recorded in CDCl_3_ using a JOEL 600 spectrometer (JOEL, USA). Chemical shifts were referenced relative to an internal standard (TMS, δH: 0.00 ppm).

## Electronic supplementary material

Supplementary Information
