# Supplementary material for: Intertidal marine sediment harbours Actinobacteria with promising bioactive and biosynthetic potential
Source: Sci Rep. 2017 Aug 30;7:10041. doi: 10.1038/s41598-017-09672-6 (PMC5577230; doi:10.1038/s41598-017-09672-6)
Supplement: Supplementary file 1 — Supplementary Information [file 41598_2017_9672_MOESM1_ESM.pdf]

Intertidal marine sediment harbours Actinobacteria with promising bioactive and biosynthetic potential

Authors: Polpass Arul Jose[1*], Bhavanath Jha[1.2*]

[1]Marine Biotechnology and Ecology Division, CSIR- Central Salt and Marine Chemicals Research Institute, G. B. Marg, Bhavnagar - 364002, Gujarat, India.

[2]Academy of Scientific and Innovative Research (AcSIR), Council of Scientific and Industrial Research (CSIR), New Delhi, India.

***Correspondence:**
Tel: +91-278-2567760 Ext. 6260; Fax: +91-278-2567562; e-mails: arulmku@gmail.com (PAJ) and bjha@csmcri.org (BJ)

Supplementary Tables

Table S1. Summary of NRPS and PKS positive isolates among the distinct actinobacterial isolates affiliated to different genus.

Genus	No. Isolates	Positive		Negative		Positive for both	Negative for Both
		NRPS	PKS	NRPS	PKS		
Glycomyces	1	1	0	0	1	0	0
Micromonospora	8	6	0	2	8	0	2
Nocardia	1	1	0	0	1	0	0
Nocardiopsis	8	6	2	2	6	1	1
Saccharomonospora	5	2	2	3	3	0	2
Streptomyces	37	24	23	13	14	18	7
Actinomadura	2	2	1	0	1	1	0
Total	62	42	28	20	34	20	12

Table S2. List of bacterial strains used in antibacterial activity screening, with their resistance pattern.

Bacteria	Culture types* (ATCC/Clinical)	Antibiotics**, and resistance profiles								
		GEN/HLG	K	CIP	E	VA	IPM	AZM	COT	CD
Bacillus subtilis	ATCC 6051	-	-	-	-	-	-	-	-	-
Enterococcus spp	Clinical isolate	R	R	S	R	R	S	-	R	-
Staphylococcus aureus	ATCC 25923	S	S	S	I	I	S	I	S	S
Escherichia coli	ATCC 8739	I	I	S	R	R	I	-	S	-
Klebsiella pneumoniae	Clinical isolate	R	R	R	R	R	R	-	R	-

*Culture types: ATCC cultures were purchased from American Type Culture Collection; Clinical strains were kindly provided by the Department of Microbiology, Government Medical College, Bhavnagar, Gujarat, India.
**Name of antibiotics: Gentamicin (GEN/HLG), Kanamycin: (K), Ciprofloxacin (CIP), Erythromycin (E), Vancomycin (VA), Imipenem (IPM), Azithromycin (AZM), Co-Trimoxazole (COT) and Clindamycin (CD). Resistance pattern was defined according to CLSI standards. R: Resistant, I: Intermediate resistant, S: susceptible, -: Not applicable.

Table S3. Summary of Blastx results: closest match, E-value, and percentage similarity of 11 unique inserts that specify PKS-II and NRPS.

Strain ID	Clone ID	Closest hit		E value	Similarity (%)
		Accession	Description (Source)		
JJ06	1N	WP051307660	Non-ribosomal peptide synthetase (*Streptomyces* sp. CNS606, isolated from marine source)	9e-61	82
JJ06	7N	SCL23407	Amino acid adenylation domain-containing protein (*Micromonospora pallida* DSM 43817 from soil)	9e-95	90
JJ23	51N	ALV82384	CDA peptide synthetase II - Condensation domain (*Streptomyces rochei* Sal35 from forest soil)	4e-89	99
JJ66	70N	EST37752	Hypothetical protein N566-11320, partial (*Streptomycetaceae* bacterium MP113-05, from marine sponge)	2e-61	83
JJ133	2N	SCE50398	Amino acid adenylation domain-containing protein (*Streptomyces* sp. di188)	3e-62	82
JJ142	131N	WP030989078	Non-ribosomal peptide synthetase (*Streptomyces* sp. NRRL S-1813, from mountain soil)	1e-59	73
JJ23	113P	AKN79417	Polyketide synthase II (*Streptomyces* sp. SCAU5110, from liquorice)	2e -79	97
JJ24	110P	AKN79418	Polyketide synthase II (*Streptomyces* sp. SCAU5133, from liquorice)	3e-78	97
JJ54	25P	AKN79344	Polyketide synthase II (*Streptomyces* sp. SCAU5027, from liquorice)	3e-82	99
JJ40	13P	ADC98315	ketosynthase alpha (*Streptomyces* sp. JAJ-06, from marine solar saltern)	1e-78	98
JJ142	142P	AGG43888	putative type II PKS-ketosynthase alpha subunit (Uncultured bacterium)	6e-75	91

Table S4. List of media and their composition used in this study for the isolation of Actinobacteria from intertidal marine sediments.

S. No	Media	Composition*
01	M1	Soluble starch 1.0%, yeast extract 0.4%, peptone 0.2% and agar 1.8%.
02	M2	Glycerol 0.6%, L-asparagine 0.1%, K_2HPO_4 0.1%, $MgSO_4.7H_2O$ 0.05% and agar 1.8%.
03	M3	Glucose 0.6%, chitin 0.2% and agar 1.8%.
04	M4	Glucose 1.0%, peptone 0.5%, malt extract 0.03%, yeast extract 0.03% and agar 1.8%.
05	M5	Chitin 0.2% and agar 1.8%.
06	M6	Sodium caseinate 0.2%, L-asparagine 0.01%, sodium propionate 0.4%, K_2HPO_4 0.05%, $MgSO_4.7H_2O$ 0.01%, $FeSO_4{\cdot}7H_2O$ 0.0001% and agar 1.8%.
07	M7	Soluble starch 1.0%, yeast extract 0.4%, $(NH_4)_2SO_4$ 0.2%, K_2HPO_4 0.1%, $MgSO_4{\cdot}7H_2O$ 0.1%, $CaCO_3$ 0.1% and agar 1.8%.
08	M8	Soluble starch 1.0%, casein 0.03%, KNO_3 0.2%, $MgSO_4{\cdot}7H_2O$ 0.005%, $FeSO_4{\cdot}7H_2O$ 0.001%, K_2HPO_4 0.2%, $CaCO_3$ 0.002% and agar 1.8%.

*All the media were prepared in 70% sea water, and amended with nalidixic acid (50 µg/ml) and cyclohexamide (100 µg/ml). All media components are in "w/v".

Supplementary Figures

Figure S1

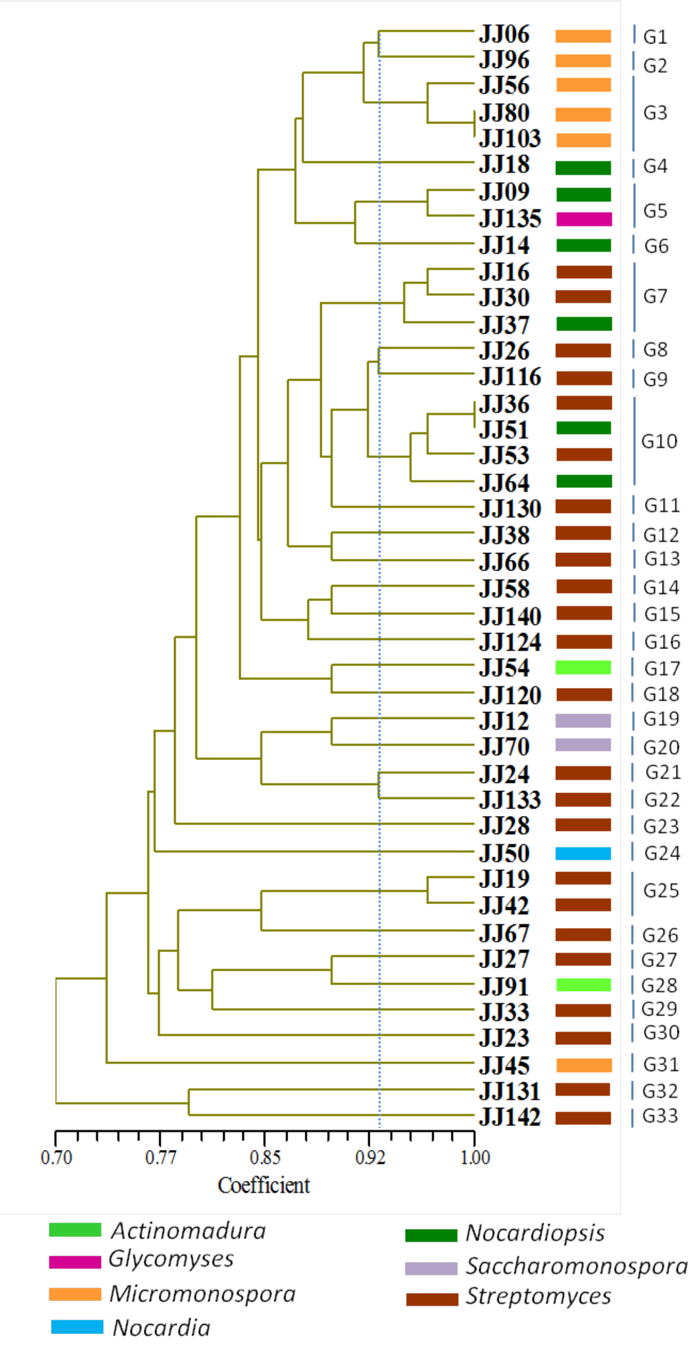

Figure S1. UPGMA dendrogram inferred by restriction pattern of amplified NRPS fragments digested by *Alu*I. The gel was analyzed by densitometry and the bands whose areas were greater than 5% of the whole lane area were used for generating binary matrix. The similarities were calculated using the Jaccard's coefficient, and the clustering was done by using the unweighted pair group method. Grouping of actinobacterial strains is showed at similarity coefficient 0.93.

Figure S2

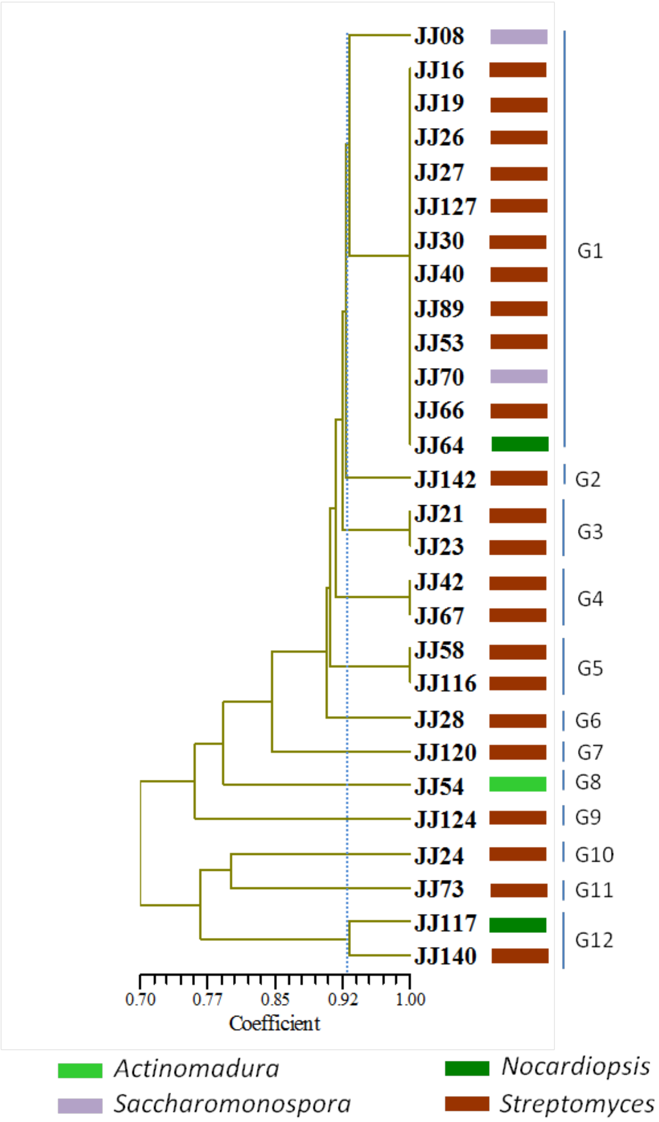

Figure S2. UPGMA dendrogram inferred by restriction pattern of amplified PKS-II fragments digested by *Alu*I. The gels were analyzed by densitometry and the bands whose areas were greater than 5% of the whole lane area were used for generating binary matrix. The similarities were calculated using the Jaccard's coeffcient, and the clustering was done by using the unweighted pair group method. Grouping of actinobacterial strains is showed at similarity coefficient 0.93.

Figure S3

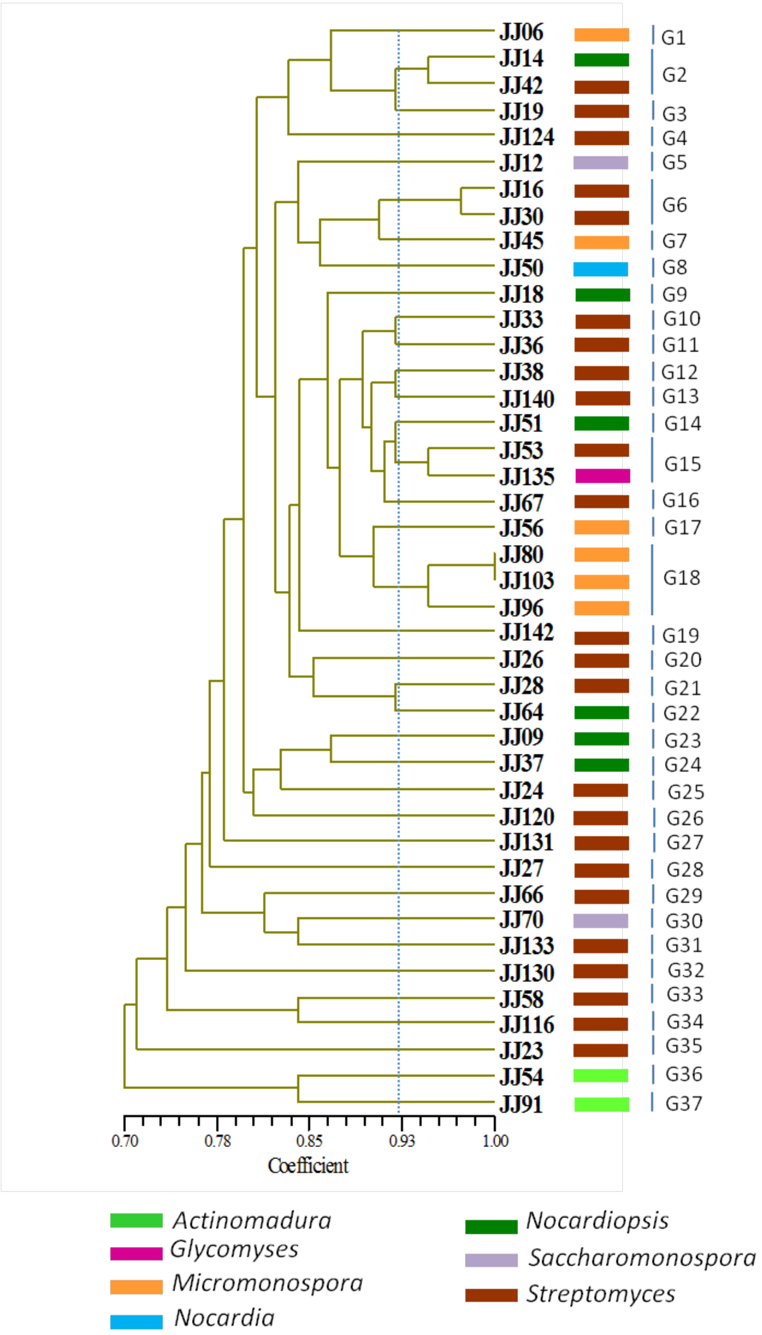

Figure S3. UPGMA dendrogram inferred by restriction pattern of amplified NRPS fragments digested by *Hae*III. The gel was analyzed by densitometry and the bands whose areas were greater than 5% of the whole lane area were used for generating binary matrix. The similarities were calculated using the Jaccard's coefficient, and the clustering was done by using the unweighted pair group method. Grouping of actinobacterial strains is showed at similarity coefficient 0.93.

Figure S4

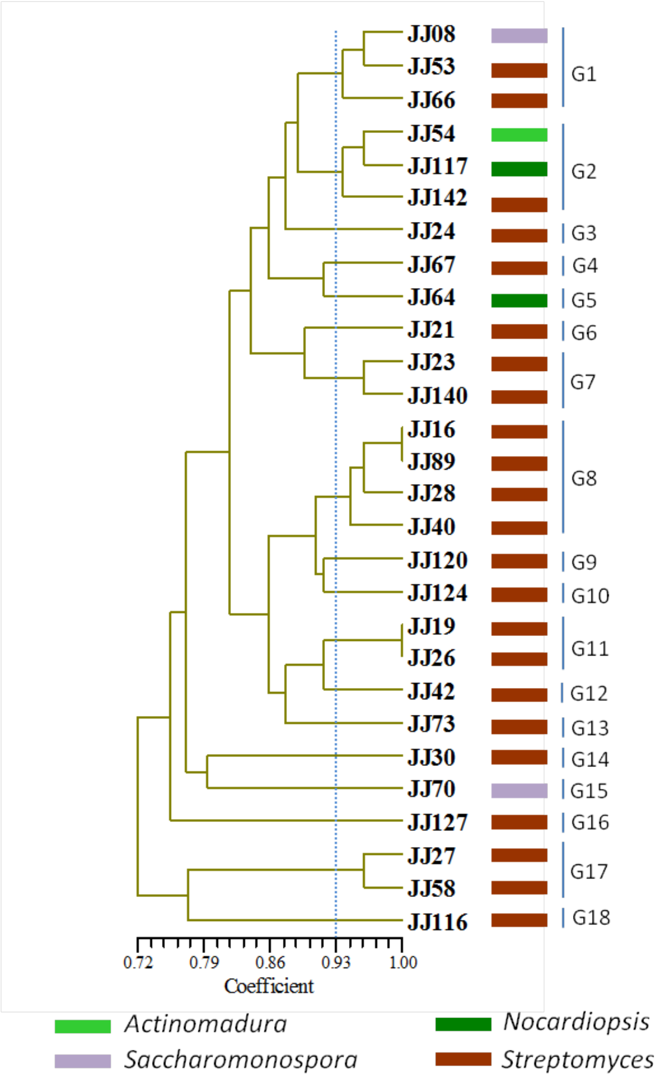

Figure S4. UPGMA dendrogram inferred by restriction pattern of amplified PKS-II fragments digested by *Hae*III. The gels were analyzed by densitometry and the bands whose areas were greater than 5% of the whole lane area were used for generating binary matrix. The similarities were calculated using the Jaccard's coeffcient, and the clustering was done by using the unweighted pair group method. Grouping of actinobacterial strains is showed at similarity coefficient 0.93.

Figure S5

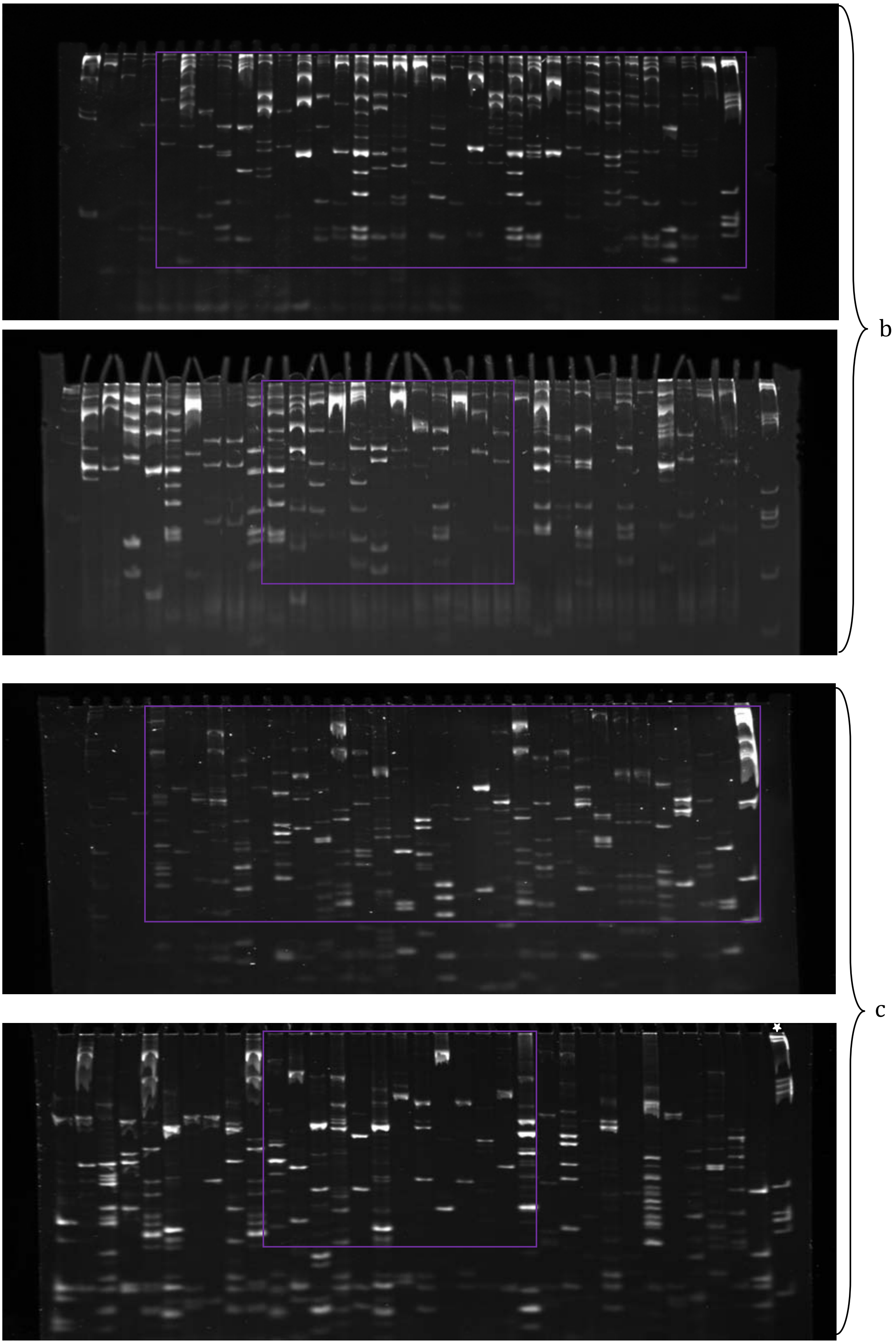

Figure S5. Original gels of Figure 7: Restriction pattern of NRPS amplicons digested by *Hae*III (b) and *Alu*I (c). *digested by *Hae*III.

Figure S6

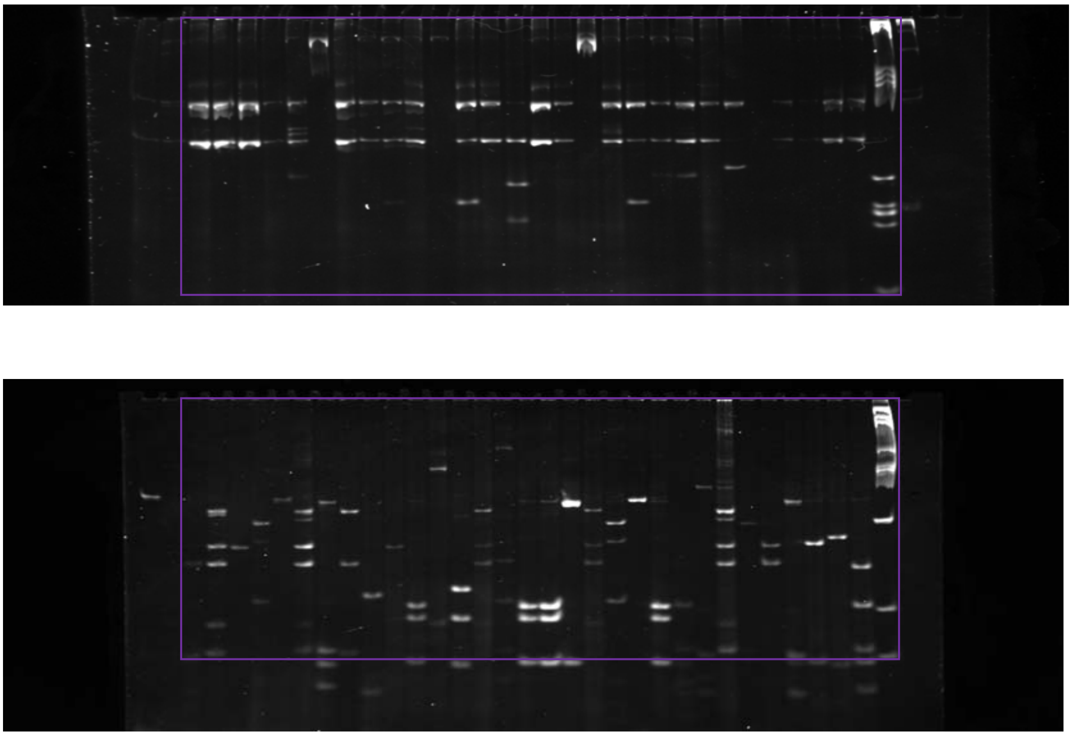

b

c

Figure S6. Original gels of Figure 8: Restriction pattern of PKS-II amplicons digested by *Hae*III (b) and *Alu*I (c).

Figure S7

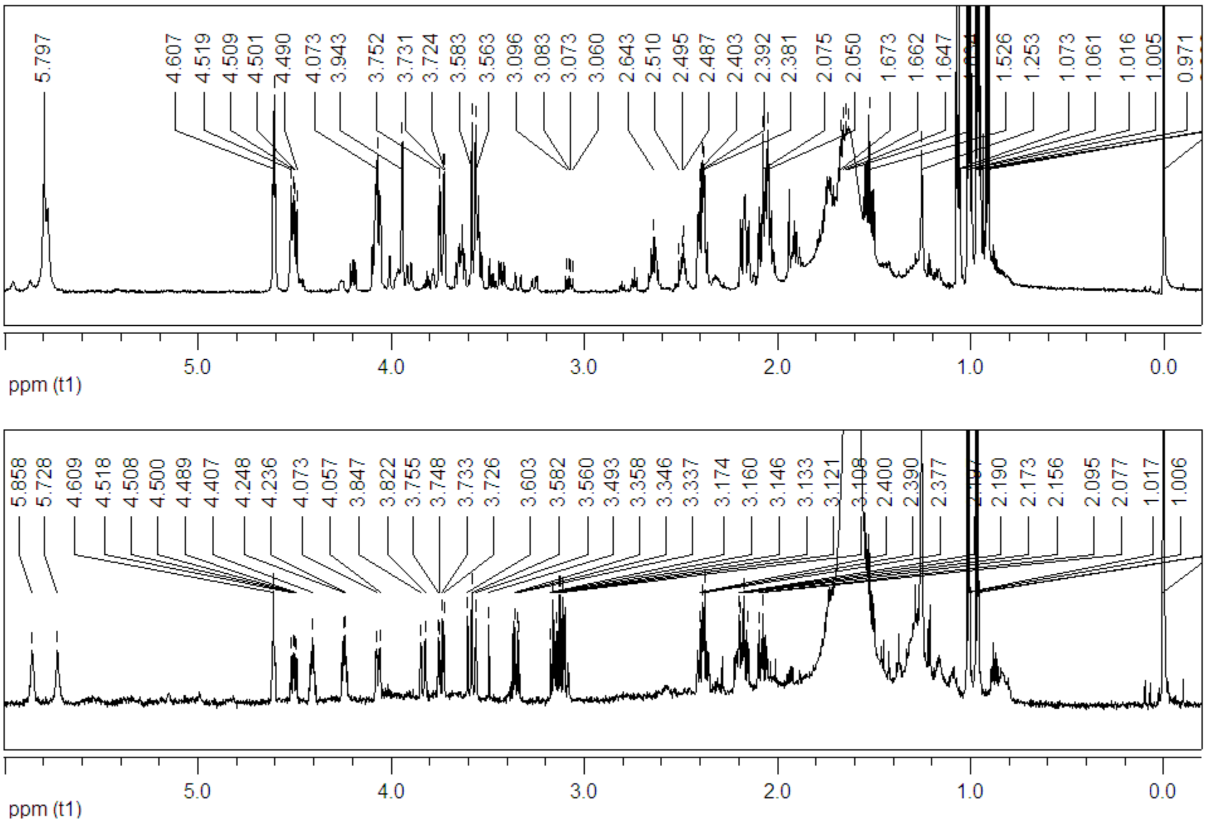

Figure S7. ^{1}H NMR spectra of compounds OBLC 02 and 03

^{1}H NMR data of compounds OBLC 02 and 03

OBLC 02: ^{1}H NMR (CDCl3, 600 MHz); δ = 4.61 (s, 1H), 4.52-4.49 (t, 2H), 4.10-4.06 (m, 2H), 3.94 (bs, 2H), 3.75-3.72 (dd, 1H), 3.67-3.62 (m, 1H), 3.58-3.55(t, 2H), 2.66-2.63 (t, 1H), 2.51-2.49 (t, 1H), 2.41-2.37 (dd, 2H), 2.19-2.15 (t, 2H), 2.10-2.01 (m, 3H), 1.07-1.06 (d, 3H), 1.02-0.99 (d, 3H), 0.97-0.95 (d, 3H), 0.93-0.91 (d, 3H).
OBLC 03: ^{1}H NMR (CDCl3, 600 MHz); δ = 5.85, 5.71 (bs, 1H), 4.60-4.59 (t, 1H), 4.50-4.48 (dd, 1H), 4.41-4.38 (m, 1H), 4.24-4.22 (t, 1H), 4.07-4.04 (dd, 1H), 3.83-3.81 (d, 1H), 3.74 - 3.71 (dd, 1H), 3.59-3.55 (t, 1H), 3.35-3.32 (dd, 1H), 3.16-3.06 (m, 2H), 2.40-2.34 (m, 1H), 2.21-2.14 (m, 2H), 2.09-2.04 (m, 1H), 1.24 (s, 2H, 1.00-0.95 (dd, 3H).
